# LPS-Stressed Bovine Endometrial Cells upon Morulae in a Transwell Model of Embryo—Maternal Talk

**DOI:** 10.3390/ani16010038

**Published:** 2025-12-23

**Authors:** Anna Lange-Consiglio, Giulia Gaspari, Paola Gagni, Giampaolo Bosi, Pietro Riccaboni, Fausto Cremonesi

**Affiliations:** 1 Laboratory of Reproduction of University Veterinary Hospital, Department of Veterinary Medicine and Animal Science (DIVAS), University of Milan, 26900 Lodi, Italyfausto.cremonesi@unimi.it (F.C.); 2 Istituto di Scienze e Tecnologie Chimiche “Giulio Natta” (SCITEC), Consiglio Nazionale delle Ricerche (CNR), 20162 Milan, Italy; paola.gagni@cnr.it; 3 Department of Veterinary Medicine and Animal Science (DIVAS), University of Milan, 26900 Lodi, Italy; giampaolo.bosi@unimi.it (G.B.);

**Keywords:** bovine, epithelial endometrial cells, LPS, embryo development, extracellular vesicles

## Abstract

During the preimplantation period, the nutrition of the embryo is dependent on luminal secretions of the uterus, which can be modified by the health status of the animal. The aim of this study was to mimic the paracrine communication between healthy or LPS-stressed epithelial endometrial cells (EECs) and embryos using aa transwell plate. The rate of in vitro embryo production, size, and concentration of extracellular vesicles (EVs), and level of secretion of Galectin-9 (Gal-9) and leukaemia inhibitory factor (LIF) were detected. Embryos were produced with an established protocol of oocyte in vitro maturation (IVM), in vitro fertilization (IVF), and in vitro embryo culture (IVC). On day 55 of IVC, one hour before the transfer of morulae in the basolateral compartment of the transwell, EECs were treated with 10 ng/mL of LPS, and IVC was continued until the eleventh day. Extracellular vesicles (EVs) were obtained from IVC medium by ultracentrifugation. Levels of Gal-9 and LIF were evaluated by ELISA. On day 7, the results did not show statistically different blastocyst rates between EECs+Embryo and EECs+LPS+Embryo (34.94 ± 1.95% and 33.06 ± 3.08%, respectively). On day 11, the rate of hatched blastocysts was 23.03 ± 3.18% in EECs+Embryo, while in EECs+LPS+Embryo, no hatching was observed. Nanosight revealed higher values in EV size and concentration in EECs+LPS+Embryo medium compared to EECs+Embryo (*p* < 0.05). In LPS-treated samples, there was a significant decrease in Gal-9 levels and a significant increase in LIF secretions compared with non-non-LPS-treated samples (*p* < 0.05). These results highlight how bidirectional secretions between EECs and embryos, crucial for embryo development, can be affected by endometritis.

## Simple Summary

Endometritis causes preimplantation embryonic loss due to altered uterine nutrient composition. This study simulated a uterine inflammatory process stressing bovine endometrial cells by bacterial lipopolysaccharide with the aim of evaluating the effects of endometritis in early embryo development by using an in vitro model that allows communication between cells and embryos by porous inserts. This disposable insert permits simultaneous culture and release of soluble molecules and extracellular vesicles in a bidirectional way between cells and embryos. Up to day 7, no differences were observed in the embryo between stressed cells and non-stressed cells. From day 9, the percentage of embryos decreased significantly (24.56% in stressed cells versus 36.84% in non-stressed), and on day 11, no hatched embryos were obtained in stressed cells compared to control cells. In addition, the evaluation of the extracellular vesicles secreted by both cells and embryos, in terms of quantity and size, highlights how the communication between cells and embryos is altered by the inflammatory environment. This study highlights how any inflammation can alter embryonic development. The evaluation of secreted extracellular vesicles could constitute a new methodological platform and provide key mechanistic data for understanding early embryonic failure caused by uterine inflammation.

## 1. Introduction

In cattle, 5 days after fertilization, the morula-stage embryo enters the uterine horn ipsilateral to the ovary from which ovulation occurred, interacting with the maternal environment via paracrine signals until implantation occurs [1,2]. Communication is regulated by molecules secreted by both the embryo and the mother. This defines a bidirectional communication system [2] that is controlled by molecules secreted in the extracellular environment or transported to target cells via extracellular vesicles (EVs) [3]. The latter are membrane nanoparticles, delimited by a phospholipid bilayer, released into the extracellular environment by a donor cell and internalized by the recipient cell [4,5,6]. Almost all cell populations secrete EVs into the extracellular environment [7,8], but in the case of maternal––embryonic communication, EVs transport molecules involved in triggering endometrial receptivity and of considerable importance for embryonic development [9].

Most studies in bovine and human species have demonstrated that endometrial-derived EVs are primarily released from the luminal epithelium, rather than the stromal compartment [10]. In healthy status, epithelial cells secrete a variety of molecules that prepare the endometrium for receptivity of the embryo and provide essential factors to the conceptus prior to and during implantation [11,12,13].

Among the pathologies that affect the reproductive cycle of dairy cows, endometritis causes early embryonic loss in the post-partum period [14], with a total frequency of embryo loss of 25–40% between day 0 and day 7 [15,16]. Early embryonic losses happen before embryo implantation occurs [17]. Implantation is a process tightly regulated by the presence of cytokines; it is necessary for endometrial tissue remodeling and to promote immune tolerance toward the developing embryos. When endometritis occurs, an imbalance in the gene expression of cytokines in the uterus can lead to endometrial cell damage and, consequently, alter maternal––embryo communication, contributing to embryonic loss and pregnancy failure [18,19]. Indeed, inflammatory molecules can be transferred via EVs from endometrial epithelial cells into the uterine microenvironment, into adjacent recipient cells to modulate local immune responses [10], and then into the embryo.

In vivo endometritis caused by LPS of *Escherichia coli* negatively affects the histotrophic composition, leading to reduced development and lower embryo quality [20]. Indeed, the resulting inflammation leads to the formation of a uterine environment hostile to embryonic development.

Studies have shown the involvement of Galectin-9 (Gal-9) in inflammatory diseases, making it a valuable biomarker for investigating this condition [21,22]. Likewise, leukaemia inhibitory factor (LIF), a key determining molecule in the development and implantation of embryos [23], represents another effective biomarker for the evaluation of embryo growth.

In this context, the aim of this study was to evaluate the effects of endometritis on early embryonic development using an in vitro model. The effects of LPS are studied directly on endometrial epithelial cells and indirectly on the embryo from the fifth day of in vitro culture. This stage is estimated to be the moment that the in vivo bovine embryo reaches the uterine cavity and comes into contact with the secretions of the endometrial epithelium. The rate of blastocyst development at different stages, detection of Gal-9, and LIF level by ELISA, along with isolation and characterization of EVs from the co-culture medium of different experimental conditions, were carried out.

## 2. Materials and Methods

Reagents

All reagents were purchased from Merck (Milan, Italy), while test tubes and culture plates were purchased from Euroclone (Milan, Italy).

Experimental design

**Figure 1:**
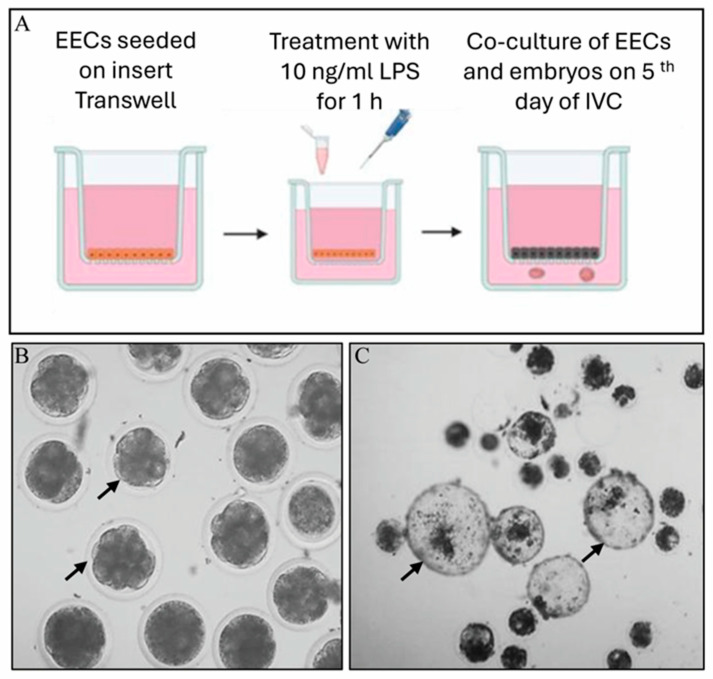
Representative scheme of EEC–embryo–embryo co-culture. (**A**) Seeding of EECs, treatment with LPS and co-culture with morulae (BioRender.com). (**B**) Arrows indicate in vitro-produced bovine morulae obtained on the fifth day of development (the morula size is about 120–130 µm). (**C**) Arrows indicate in vitro-produced bovine embryos on the eleventh day of development (about 180–200 µm).

Communication between EECs and embryos was carried out using a transwell system, where EECs were seeded in the apical compartment and embryos in the basolateral compartment in a low-tension oxygen environment. Communication between EECs and embryos was carried out using a transwell system, where EECs were seeded in the apical compartment and embryos in the basolateral compartment in a low-tension oxygen environment. Only EECs were directly stressed by LPS, and this effect was indirectly evaluated by embryo development. Only EECs were directly stressed by LPS, and this effect was indirectly evaluated by embryo development. The trial design tested the following experiments: (i) culture of EECs in in vitro culture medium [(IVC, used for embryo culture), Stroebech Media, Odense, Denmark)] and HG-DMEM (EECs); (ii) culture of EECs treated with LPS at the concentration of 10 ng/mL for 1 h (EECs+LPS); (iii) culture of embryos for 7, 9, and 11 days (Embryo); (iv) co-culture of EECs seeded on apical compartment of transwell insert and morulae transferred in the basolateral compartment of transwell plate at 5 days of embryo culture (EECs+Embryo); (v) co-culture of EECs and morulae after LPS treatment of EECs (EECs+LPS+Embryo) (Figure 1).

To set up this system, EEC culture on a transwell insert was established and evaluated by carrying out two experiments:

Experiment 1. EEC culture on standard plates. EECs were plated in flask with in vitro culture medium (IVC) and incubated at 38.5 °C with 5% CO_2_ and low oxygen tension (5%). Morphology and viability were evaluated. Control was carried out with Dulbecco’s Modified Eagle’s Medium high glucose (HG-DMEM) in 5% CO_2_.

Experiment 2. EEC culture on transwell system. EECs were plated on the 0.4 µm insert of the transwell apical portion, and the formation of the monolayer was evaluated by trans-epithelial electrical resistance (TEER) and histological analysis, both in IVC medium and HG-DMEM in an incubator at 38.5 °C with 5% CO_2_ and 5% O_2_.

### 2.1. Uteri Collection and Endometrial Cell Isolation

Fresh bovine uteri were collected from cows slaughtered for human consumption and unrelated to our experiments. Endometrial samples were obtained from 3 different healthy normal-cycling cows at the diestrus stage with an obvious corpus luteum on the ovaries (middle––late luteal phase) and processed within two hours of slaughter.

Endometrial cells were obtained according to the protocol described by Donofrio et al. [24]. Briefly, the endometrium fragments were digested in 25 mL of sterile filtered Hanks’ buffered salt solution supplemented with 50 mg collagenase II (Merck catalog n° CII-bioc), 100 mg bovine serum albumin, and 10 mg DNase I (Merck, catalog n° 11284932001) for 90 minutes at 38.5 °C in a shaking bath. Then, cells were filtered with an 80 μm filter, centrifuged at 300× *g* for 10 minutes, and washed twice in phosphate buffer solution. Before seeding, the total number of viable cells was evaluated by the exclusion method, staining with trypan blue and using a Bürker chamber (Securlab, Roma, Italy).

### 2.2. Endometrial Epithelial Cell (EEC) Separation

A pool of endometrial cells obtained from the three uteri was established in HG-DMEM supplemented with 10% fetal bovine serum (FBS), 100 UI/mL penicillin–100 μg/mL streptomycin, 0.25 mg/mL amphotericin B, and 2 mM L-glutamine (complete medium). After 18 h of incubation, endometrial stromal cells were adherent to the bottom of the flask, while endometrial epithelial cells remained in suspension. So, at this time, the culture medium rich in epithelial cells (EECs) was removed and seeded again. In this way, stromal and EECs were obtained separately. For maintenance of cultures, EECs were plated in 75 cm^2^ flasks up to 1 × 10^5^ cells/cm^2^ and incubated at 38.5 °C with 5% CO_2_ in a humidified atmosphere. Once sub-confluency was reached (80%), both cell populations were detached using 0.05% trypsin––EDTA and then many vials of cells were cryopreserved at −196 °C at passage 1 with 10% dimethyl sulfoxide and 90% FBS.

For each study and replicate sample, the same pool of EECs at passage 1 (P1) was used after thawing. All experiments were carried out using the same cell line previously isolated at P1.

### 2.3. Experiment 1: EEC Culture in Standard Plates

(a) EEC Morphology

Thawed EECs at P1 were seeded on coverslips 24 × 24 mm positioned in different 6-well plates with a density of 50,000 cells/well in three different replicates. Some plates were incubated with HG-DMEM complete medium at 38.5 °C in a humidified atmosphere with 5% CO_2_. Other plates were incubated with IVC medium that does not contain FBS but synthetic serum replacement and human albumin in low concentration. Incubation was performed at 38.5 °C in a humidified atmosphere with 5% CO_2_ and 5% O_2_. Cells were cultured for 11 days in HG-DMEM and in IVC medium, receiving media changes every three days. Cells were observed with a phase contrast microscope by May––Grünwald–Giemsa staining.

(b) EEC viability by MTT test

Thawed EECs at P1 were seeded in 12-well plates with a density of 15,000 cells/well and cultured for 11 days in HG-DMEM and in IVC media. Cells were compared for their viability by the MTT test in three different replicates.

This assay is based on the reduction of the yellow tetrazolium dye 3-(4,5-dimethylthiazol-2-yl)-2,5-diphenyltetrazolium bromide (MTT) to a purple water-insoluble formazan in cells. On the fourth, eighth, and eleventh day of the culture period, the medium was discarded, and MTT solution was added for 2 h and incubated at 38.5 °C. The medium was discarded, and the resulting formazan crystals were solubilized with a mixture of HCl 1N//isopropanol (1:24) on a heated shaker.

The absorbance of the resulting solutions was read at a wavelength of 595 nm with a spectrophotometer Onda V-10 Plus (VIS; Sinergica, Milan, Italy). Viability results are expressed as a percentage of control. Cell seeding number, MTT concentration, and incubation time of reaction were standardized to limit changes due to these variables [25].

### 2.4. Experiment 2: EEC Culture on Transwell System

Based on the results of the previous experiments for all experimental conditions, EECs were cultured with IVC medium. Epithelial endometrial cells (15,000 cells/insert) were seeded in the apical portion of a transwell system with 0.5 mL of IVC medium, while 1.5 mL of IVC medium was added in the basolateral portion. These plates were incubated at 38.5 °C under 5% CO_2_ and 5% O_2_.

The formation of the EEC monolayer on inserts was evaluated by trans-epithelial electrical resistance (TEER) and histological analysis in three different replicates.

(a) TEER Test

The TEER is a quantitative measure of the barrier integrity [26] examined using an EVOM2 Epithelial Voltmeter with STX3 electrode (World Precision Instrument, Hertfordshire, United Kingdom). The readings were performed on three wells (of 12-well plates) with cell monolayers and on a control well in the absence of cells. The formation of barriers was monitored from one to eleven days of EEC culture.

For electrical measurements, two electrodes were used: one placed in the upper compartment and the other in the lower compartment, separated by the cellular monolayer. The system has a measurement range of 1–9999 Ω, with 1 Ω resolution. Each stick of the electrode pair (4 mm wide and 1 mm thick) contains a silver/silver chloride pellet for measuring voltage and a silver electrode for passing current.

The measurement procedure includes measuring the blank resistance (RBLANK) of the semipermeable membrane only (without cells) and measuring the resistance across the cell layer on the semipermeable membrane (RTOTAL). The cell-specific resistance (RTISSUE), in units of Ω, can be obtained as reported by Srinivasan et al. [27]:

$$R_{TISSUE}\left(Ω\right)=R_{TOTAL}-R_{BLANK}$$

TEER values are typically reported (TEERREPORTED) in units of Ω/cm^2^ and calculated as:

$${TEER}_{REPORTED}=R_{TISSUE}\left(Ω\right)-M_{AREA}({cm}^{2})$$

(b) Histological analysis of EECs on a transwell insert

Transwell inserts were fixed in 10% formalin, removed from the plastic of the 6-well plate, dehydrated via graded alcohol passages, and embedded in paraffin wax according to Manna and Caradonna [28]. Histological sections of 5–7 µm were obtained from the paraffin blocks and mounted on slides for hematoxylin and eosin staining and microscopic observation.

### 2.5. In Vitro Embryo Production

The in vitro embryo production was organized in three steps: in vitro maturation (IVM), in vitro fertilization (IVF), and in vitro embryo culture (IVC), and the experiment was replicated three times.

In vitro maturation: using an 18 G needle, follicular fluid aspirated from the ovaries was collected in Falcon tubes in a water bath at 37 °C. After 10 min, the pellet formed at the bottom of the Falcon tube was transferred to a Petri dish to select cumulus oocyte complexes (COCs) using a stereomicroscope. Selected COCs were washed three times in oocyte-washed medium (Stroebech), once in IVM medium (Stroebech), and incubated in IVM medium for 24 h at 38 °C with 5% CO_2_. Oocyte maturation was performed in 4-well plates with 0.5 mL of IVM medium with up to 45 COCs/well.

In vitro fertilization: frozen—thawed semen of a single bull of proven fertility was thawed in a water bath at 35 °C for 30 s and centrifuged for 5 min at 300× *g* with 4 mL of semen wash medium (Stroebech). The supernatant was discarded, and the remaining semen pellet was resuspended in 2 mL of semen wash medium and centrifuged again at 300× *g* for 5 min. The supernatant was discarded, leaving 400 μL of pellet. The count of spermatozoa was performed by a Mackler chamber, and the final concentration was adjusted to 1 × 10^6^ sperm/mL. At the end of maturation, fertilization was performed for 18 h, after washing all COCs in IVF medium (Stroebech).

For IVC, at the end of gamete co-culture, cumulus cells were mechanically and completely removed from presumptive zygotes that were transferred into 4 wells (up to 30 zygotes per well) with 500 µL of IVC medium (Stroebech) and 400 µL of mineral oil (Stroebech). In vitro culture was performed for 5 days in an incubator with 5% O_2_, 5% CO_2_ and 90% N_2_ in a humidified atmosphere at 38.5 °C.

All embryo productions were performed in three replicates.

### 2.6. Effect of LPS on Embryo Development

Cryopreserved EECs at passage 1, obtained from a pool of three different uteri previously described, were thawed the day of IVF and seeded on a transwell membrane. One hour before morulae transfer, EECs were treated with 10 ng/mL of LPS (Merck, catalog n° 437627) for 1 h, washed with new IVC medium and cultured again with 0.5 mL of IVC medium (Figure 1A). On the fifth day of IVC, only morulae were selected and transferred to the basolateral compartment of the transwell with 1.5 mL of IVC medium (60 morulae/well; Figure 1B). Transwells with EECs and morulae were incubated until the eleventh day in an incubator with a low tension of oxygen.

The fifth day of embryo development coincides with the fifth day of EEC culture.

### 2.7. Embryo Rate Evaluation

On day 7 (48 h post LPS treatment), day 9 (96 h post LPS treatment), and day 11 (6 days post LPS treatment) of embryo culture, the number of blastocysts, expanded blastocysts, and hatched blastocysts were counted by visualization under a stereomicroscope.

### 2.8. Evaluation of Galectin-9 and LIF by ELISA

On days 7 and 11 of culture, all culture medium was collected from apical and basolateral portions of each plate of each experimental condition to estimate soluble Gal-9 levels (bovine Gal-9 ELISA, cat. no. BODL00059, AssayGenie, Dublin, Ireland) and LIF level (bovine LIF ELISA Kit cat.no. A78384, Antibodies, Stockholm, Sweden) with a sandwich ELISA kit by strictly following the manufacturer’s protocol. All samples previously preserved at −80 °C were run on a single plate for each detection and in duplicate. Optical density was measured at 450 nm with a FLUOstar Optima microplate reader (BMG Labtech), Ortenberg, Germany). Soluble Gal-9 and LIF concentrations were calculated with BMG Optima 2.10 R2 software.

### 2.9. Isolation of EVs and Characterization by Nanosight, Western Blot, and Transmission Electron Microscopy

To isolate EVs, the IVC medium from the apical portion and medium deprived of embryos from the basolateral portion was collected and centrifuged at 250× *g* for 10 minutes, then at 4000× *g* for 20 minutes to eliminate cells and debris and, lastly, ultracentrifuged at 100,000× *g* at 4 °C for 1 h (Beckman Coulter OptimaX, Milan, Italy). The EV pellet was resuspended in serum-free medium. The EV pellet was resuspended in serum-free medium.

The characterization of EVs followed minimal information for studies of extracellular vesicles (MISEV guidelines) [29,30].

(a) Nanosight

The size and concentration of EVs were determined by a Nanosight NS300 system (Malvern, UK) configured with a 532 nm laser. Samples were diluted in micro-filtered phosphate buffer solution. A syringe pump ensured consistent flow during measurement. Three 60 s videos were recorded and analyzed using Malvern NTA software version 3.4.

(b) Western blotting

Isolated EVs in reductive Laemmli buffer were treated for 5 min at 95 °C. The sample was separated by SDS-PAGE (4–20%, Mini-Protean TGX Precast protein gel, Bio-Rad, Hercules, CA, USA) and transferred onto a nitrocellulose membrane (Bio-Rad, Trans-Blot Turbo). The blocking step was performed to saturate nonspecific sites for 1 h with 5% (*w*/*v*) bovine serum albumin (BSA) in TBST (tris-buffered saline: 150 mM NaCl, 20 mM Tris–HCl, pH 7.4, and 0.5% Tween 20). Membranes were incubated overnight at 4 °C with anti-TSG101 (1:1000, Santa Cruz, Biotechnology, Santa Cruz, CA, USA), anti-CD63 (1:1000; BD Pharmingen, San Jose, CA, USA), and anti-Alix (1:1000, Santa Cruz).

After washing with TBST, membranes were incubated with the horseradish peroxidase-conjugated (Jackson ImmunoResearch, West Grove, PA, USA) secondary antibodies diluted 1:3000 for 1 h. After washing, the signal was detected using Bio-Rad Clarity Western ECL Substrate (Bio-Rad) and imaged using a Chemidoc XRS+ (Bio-Rad).

(c) Transmission electron microscopy

The EVs were fixed in a mixture of 2% paraformaldehyde and 2.5% glutaraldehyde, then post-fixed in 1% OsO4 and 1.5% potassium ferrocyanide and stained with 0.5% uranyl acetate. After that, EVs were dehydrated in a graded ethanol series, infiltrated with ethanol and resin (Araldite-Epon, Mouser Electronics, Milano, Italy), then in 100% Epon, and finally, polymerized at 60 °C. Sections 70 nm thick were observed with a Zeiss LEO 912ab (Oberkochen, Germany) energy filtering transmission electron microscopy (TEM) operating at 120 kV. Digital images were acquired using a CCD-BM/1K system operating with the iTEM (Olympus Soft Imaging Solutions, software version 5.x).

### 2.10. Statistical Analysis

The nonparametric Kruskal––Wallis H-test with Dunn’s comparison or the one-way analysis of variance (ANOVA) test with Bonferroni’s correction was used for multiple comparison by Graph Pad 3. *p* < 0.05 was considered statistically significant.

## 3. Results

### 3.1. Experiment 1: EEC Culture in Standard Plates

(a) EEC morphology

**Figure 2:**
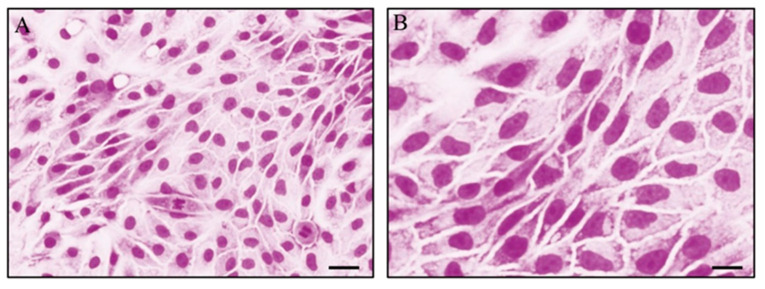
May–Grünwald–Grünwald–Gyemsa staining of EECs cultured in complete HG-DMEM (**A**) and in IVC (**B**). Different magnification to demonstrate absence of pyknotic elements of EECs cultured in IVC medium: (**A**) magnification 20×, scale bar 10 µm; (**B**) magnification 60×, scale bar 25 µm.

By microscopical analysis, cells cultured in IVC medium appear in the cuboidal form and are flat. The EECs are closely attached to one another in a single layer with minimal extracellular matrix between them. Necrotic or pyknotic cells are not present (Figure 2).

(b) EEC viability by MTT test

The DMEM culture condition was the reference point equivalent to 100%. All other points and culture conditions were compared to this single group. The MTT results were obtained by first subtracting background absorbance from all well readings. Then, the average absorbance data for each sample was measured, and finally, the value of cell viability was obtained using the formula: Viability % = 100 × (Absorbance treated IVC well)/(Absorbance control DMEM well). A high percentage indicates great vitality in terms of high cellular metabolic activity, considering DMEM 100%.

The MTT results for EEC culture in IVC medium show high viability values for each time evaluated (4, 8, and 11 days) without statistically significant differences between them. Different components of two culture media (DMEM and IVC) and the effect of serum-free culture (IVC medium) appear to be negligible on the assay measurements. Data are represented in Table 1.

### 3.2. Experiment 2: EEC Culture on Transwell System

(a) TEER test

TEER test showed an initial value of 0 Ω/cm^2^ at day 0 of culture with a continuous increase of values that arrived at 800 Ω/cm^2^ on the fifth day of EEC culture, indicating the formation of a monolayer of EECs on insert with IVC medium. This value increases to 1000 Ohm/cm^2^ until the eleventh day of the experiment. On the fifth day of culture, the monolayer is formed to start the experiment of co-culture with morulae.

(b) Histological analysis of EECs on transwell insert

**Figure 3:**
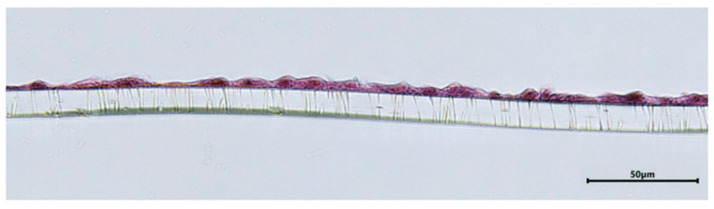
Histology by hematoxylin and eosin staining of a section of the insert, revealing the monolayer of EECs, forming a uniform tissue structure. Magnification 40×, scale bar 50 µm.

By histological section and staining with hematoxylin and eosin, it was possible to observe that the endothelial cells seeded on the transwell membrane formed a monolayer (Figure 3).

### 3.3. Effect of LPS on Embryo Development

A mean of 6 oocytes per ovary was retrieved from the 950 ovaries collected at the slaughterhouse, with a total of 1786 oocytes collected. On the fifth day of embryo culture, 1161 morulae were assigned to the three experimental conditions: Embryo, co-culture EEC+Embryo and co-culture EEC+LPS+Embryo. Embryo condition alone was performed in the same transwell system plate, but in the absence of cells. The embryo yield for each experimental condition and each time set point was calculated as a ratio between the total number of embryos obtained on the specific day (7, 9, or 11) and the number of morulae obtained on the fifth day post-IVF in five replicates (Table 2).

The embryonic development rates on the seventh day post-IVF are not statistically different between Embryo, EECs+Embryo, and EECs+LPS+Embryo (33.17% vs. 34.94% and 33.06%, respectively).

Statistically significant differences (*p* < 0.05) occurred on the ninth day of development, with a rate of 33.52% in EECs+Embryo compared with 23.98% in EECs+LPS+Embryo. On the eleventh day of development, no hatched blastocysts were found (0%) in EEC+LPS+Embryo, compared with EEC+Embryo (23.03%).

### 3.4. Evaluation of Gal-9 and LIF by ELISA

**Figure 4:**
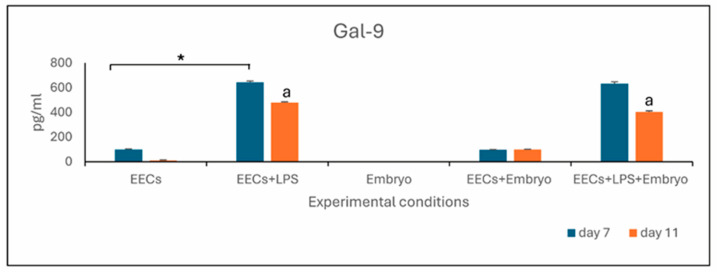
Level of Gal-9 expressed in pg/mL. Letter “a” represents statistically significant differences within the same experimental group (*p* < 0.05). Asterisk on the line represents statistically significant differences between different experimental groups (*p* < 0.05).

After treatment with LPS, there was a notable elevation of soluble Gal-9 levels in the culture medium of EECs+LPS compared with only EECs (*p* < 0.05), both on days 7 and 11. When EECs were treated with LPS, both in the presence and absence of embryos, Gal-9 levels significantly differed (*p* < 0.05) between days 7 and 11. The Gal-9 level in the embryo medium (Embryo) in the absence of epithelial cells was not detectable, while in the co-culture medium EEC+Embryo, the level of Gal-9 was measurable but unchanged with respect to the EECs (Figure 4).

After LPS treatment, the LIF level of EECs decreased in a statistically significant manner (*p* < 0.05) both on day 7 and day 11 (EECs+LPS) and also in co-culture with embryos (EECs+LPS+Embryo) compared to EECs, in which LIF increased significantly on day 11 during embryo development (Embryo) (*p* < 0.05; 7-day vs. 11-day).

**Figure 5:**
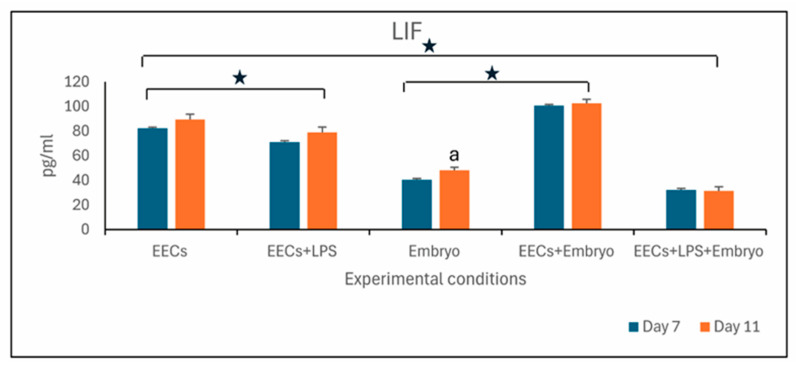
Level of LIF expressed in pg/mL. Letter “a” represents statistically significant differences within the same experimental group (*p* < 0.05). Stars on the line represent statistically significant differences between different experimental groups (*p* < 0.05).

The level of LIF was higher (*p* < 0.05) in EECs+Embryo with respect to the single cultures of Embryos (Figure 5).

### 3.5. Isolation and Characterization of EVs

**Figure 6:**
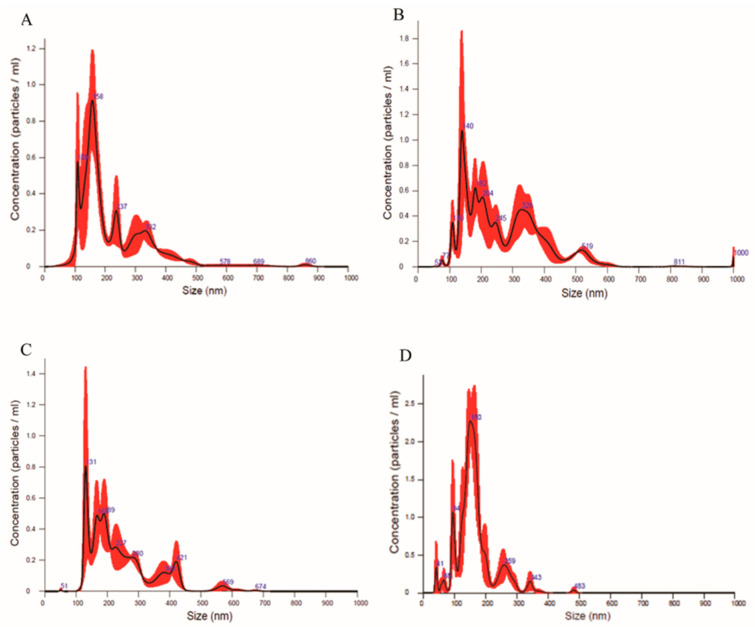
Characterization of EVs. Graphics obtained by Nanosight instruments before the final value obtained considering the dilution of sample for the lecture by the Nanosight instruments. (**A**) Results on day 7 in EECs+Embryo medium and (**B**) in EECs+LPS+Embryo. (**C**) Nanosight results on day 11 in EECs+Embryo medium and in (**D**) EECs+LPS+Embryo. EV sizes are expressed in nm and EV concentrations in particles E5/mL.

On day 7 of culture, NanoSight analyses reported that EVs isolated from the co-culture medium of EECs+Embryo had a size of 233.1 ± 20.4 nm and a concentration of 2.88 × 10^11^ ± 1.02 × 10^10^ particles/mL (Figure 6A). EVs isolated from the co-culture medium of EECs+LPS+Embryo had a size of 269.9 ± 11.0 nm and a concentration of 8.94 × 10^11^ ± 1.37 × 10^10^ particles/mL. Differences are statistically different (*p* < 0.05) both for size and concentration, with higher values in EECs+LPS+Embryo compared to EECs+Embryo (Figure 6B).

On day 11 of culture, EVs isolated from the co-culture medium EECs+Embryo had a size of 216.4 ± 13.1 nm and a concentration of 3.80 × 10^11^ ± 1.31 × 10^10^ particles/mL (Figure 6C), while EVs isolated from the co-culture medium of EECs+LPS+Embryo had a size of 240.3 ± 10 nm and a concentration of 9.6 × 10^11^ ± 1.19 × 10^10^ particles/mL (Figure 6D). Even at this time of co-culture, differences were statistically different (*p* < 0.05) both for sizes and concentrations, with higher values in EECs+LPS+Embryo compared to the EECs+Embryo.

**Figure 7:**
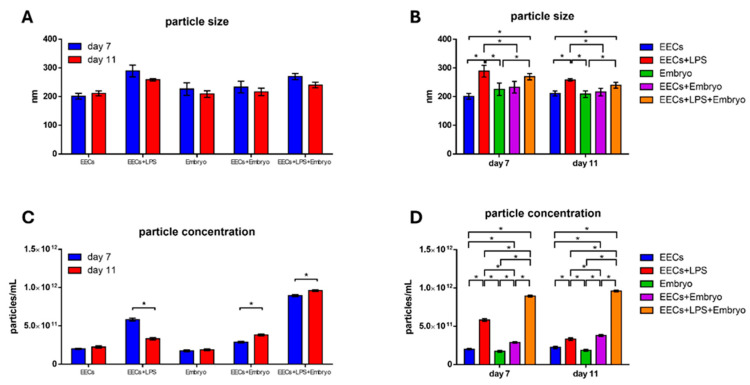
Characterization of EVs by Nanosight. (**A**) Data comparing sizes between days 7 and 11 of embryo culture period and (**B**) among different experimental conditions. (**C**) Data comparing sizes between days 7 and 11 of embryo culture period and (**D**) among different experimental conditions. Legend: lowercase asterisks represent statistically significant differences (*p* < 0.05).

In Figure 7, reported data comparing sizes and concentrations between days 7 and 11 of embryo culture period and among different experimental conditions.

**Figure 8:**
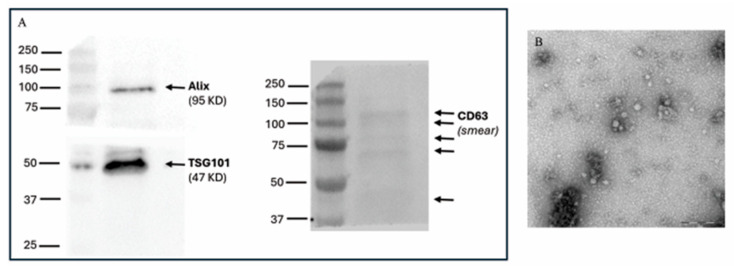
Examples of EV characterization. (**A**) Western blot for typical EV markers: CD63, Alix, and TSG101. (**B**) Transmission electron microscopy (TEM) analysis of EVs inside the embryo culture media revealed typical morphologies of vesicles (scale bar: 0.2 μm).

Western blot analysis allowed verification of the presence of specific EVs’ internal markers (Alix), and specific surface markers (CD63 and TGS101) (Figure 8A and Figures S1–S4), confirming that the preparation contained EVs.

Transmission electron microscopy confirmed the efficiency of the isolation method for EVs, as revealed by their spheroid morphology, with a moderately electron-dense coat (Figure 8B).

## 4. Discussion

This study aimed to evaluate the in vitro effects of EECs on embryo development. Cells were seeded in the apical compartment, while embryos from the fifth day of development (morula stage) were cultured in the basolateral portion. The morula stage is the moment at which in vivo bovine embryos leave the tubal environment to reach the uterine horn through the utero-tubal junction. Ideally, the porosity of the membranous support should allow the transit of molecules released by the EEC towards the embryonic culture medium and vice versa to mimic embryo––maternal communication.

Many researchers have studied endometrial cells stressed by LPS [15,18,31,32,33]. In this study, to simulate an inflammatory process, EECs were stressed using LPS (10 ng/mL for 1 h) based on our previous results [34] that highlighted this dosage as the most effective to obtain a model of endometrial inflammation. In this study, to simulate an inflammatory process, EECs were stressed using LPS (10 ng/mL for 1 h) based on our previous results [34] that highlighted this dosage as the most effective to obtain a model of endometrial inflammation.

The culture of EECs and embryos (only from the fifth day of development) had to take place simultaneously in the transwell plate, using a specific embryo culture medium (IVC) in the basal compartment (then in contact with EECs) and with low oxygen tension (5%). For this, at first, it was essential to test possible negative effects of IVC medium and low-tension oxygen on the growth of EECs that are usually cultured in HG-DMEM medium with 5% CO_2_ and atmospheric oxygen tension. In vitro culture medium was also chosen over DMEM because it does not contain fetal calf serum or EVs. These two different culture conditions equally supported the growth of EECs, which were seeded on the apical portion of transwell insert and developed in a histologically verified monolayer.

TEER measurement, monitored from day 0 of culture until when embryos were added to the basolateral compartment of the culture system, demonstrated the formation of an epithelial barrier that was maintained until the end of the culture.

The culture of EECs in IVC medium, which differs from DMEM in its composition, particularly in the absence of serum, may have influenced MTT assay readings by modulating cell biological responses, including metabolic activity and, consequently, MTT reduction [25]. Despite this, cell viability remained notably high. Consequently, all subsequent experiments were performed by culturing EECs in IVC medium.

Our results show that inflammatory molecules released by EECs, following treatment with LPS, require more than 48 h to induce a significant reduction in embryo yield. Indeed, the morulae did not immediately suffer the effect of stress, considering that the embryo yield at the blastocyst stage (seventh day of development) does not differ significantly between EECs+Embryo and EECs+LPS+Embryo. On the contrary, on the ninth day of development, a progressive and statistically significant decrease in embryo yield was registered in the EECs+LPS+Embryo compared with EECs+Embryo. In any case, in EECs+LPS+Embryo, none of the morulae were able to support further development, and no hatched blastocysts were found. These embryos were found to be degenerate, or at a lower developmental stage than expected.

Among inflammatory molecules, Gal-9 levels in the culture medium were measured to verify the induced inflammation by LPS. Gal-9 is a lectin widely expressed in various tissues and cell types associated with the immune system. Elevated levels of circulating Gal-9 have been reported in humans infected with various viruses, bacteria, and parasites [35]. Some publications reported that Gal-9, a bidirectional immunomodulator, is exclusively expressed by the epithelial cells of the normal endometrium [36] and might be a marker of endometrial receptivity before implantation [37]. Brubel et al. [21] detected a markedly increased serum Gal-9 level in both minimal––mild and moderate–severe endometriosis when compared with healthy controls. Since Gal-9 expression becomes abundant under pathological conditions, this molecule is considered a potential biomarker for the noninvasive laboratory diagnosis of endometriosis in human medicine. The notable elevation of soluble Gal-9 levels in the culture medium of EECs+LPS+Embryo compared with EECs-Embryo strongly suggests a stressed effect of LPS on EECs, as previously demonstrated by previous literature and our research group [15,18,31,32,33,34].

The decrease in decrease in embryo development in stressed medium confirms the effect of LPS, and a correlation of embryo development to the level of LIF in the co-culture medium. The LIF is part of the interleukin-6 family and, like its receptor LIF-R, is expressed by different cells, including mouse preimplantation embryos during development from the fertilized egg to the blastocyst [38]. Our study shows an increase in LIF concentration in EECs+Embryo culture medium from the seventh to the eleventh day that could reflect a possible increase in LIF expression through embryo development. Vice versa, the lowest values of LIF level were obtained on the eleventh day in co-culture EECs+LPS+Embryo. The adverse environment due to the treatment of EECs by LPS blocked embryo development but also reduced the secretion of LIF. These data overlap with the data of Zollner et al. [39] that showed a significantly higher LIF concentration in the culture medium of embryos with a well-formed trophectoderm than poorly formed embryos, and with the data of Chen et al. [40], who noted a lower expression of LIF in arrested embryos. It seems possible that LIF has a critical role for embryo development [41,42,43], where adding LIF to the culture medium increased the rate and the quality of the blastocyst. Although the exact role of LIF in embryo development remains controversial, Li et al. [44] introduced LIF as a potential biomarker for predicting clinical pregnancy, causing significantly higher LIF concentrations in the embryo culture media of women who conceived after ART treatment compared to those who did not conceive. Also, EECs secrete LIF, but after LPS treatment, either in the presence or absence of embryos, there is a decrease in this molecule. Maternal expression of LIF is essential for implantation in the mouse. Indeed, in mice, LIF is upregulated in uterine glandular epithelium on day 4, just before implantation by action of nidatory estrogen [45]. Female mice lacking the functional LIF gene are unable to support implantation, although they produce viable blastocysts that are then transferred to the pseudopregnant mother [46].

Our study provides important information on the effects that endometrial stressed cells can have on the embryo. It is known that EVs are released by most cells and are involved in numerous physiological and pathological processes, including endometritis [7]. Through the polycarbonate membrane insert, EECs release molecules and EVs, which negatively affect the underlying embryos on the ninth and eleventh days of embryo development but not on the seventh day, presumably as occurs in vivo, a few days after embryo arrival in the uterus. The analysis of sizes and concentrations of EVs isolated from EECs+Embryo culture medium highlights how the values of these parameters are higher in the medium resulting from the treatment with LPS.

Obviously, the exchange of molecules and EVs is bidirectional; in the culture medium analyzed, there are both EEC secretions and embryo secretions. Embryonic EVs could influence the metabolic processes of EECs just as endometrial EVs could influence embryonic development.

Multiple studies have shown that embryos produce and release EVs that differ based on the developmental stage of the embryo itself, but also on sex, competence, and quality of the embryo [47,48]. This applies to both the concentration and size of the vesicles. In fact, as demonstrated by Dissanayake et al. [49], degenerating or degenerated bovine embryos release a higher concentration of EVs into the culture medium than viable embryos that have reached the blastocyst stage. Embryo-derived EVs can be internalized by endometrial cells [50,51,52], inducing transcriptomic modifications in these cells, particularly by activating interferon tau (IFNT) signaling [53]. Therefore, embryo-derived EVs may play an important role in facilitating embryo––maternal interactions during the preimplantation period [54]. Vice versa, uterine EVs are internalized by bovine embryos and modulate their development and quality. The supplementation of EVs collected from uterine fluid to the IVC medium significantly increases blastocyst [55].

In the culture media analyzed in our study, the concentration of EVs is higher after LPS stress both in EECs+LPS and EECs+LPS+Embryos, confirming the data in the literature. The higher size values could be correlated to the presence of particles of dimensions around 1000 nm that represent apoptotic bodies. In addition, it has been previously described that cells release EVs in response to stress stimuli to spread signaling molecules or to remove waste material [56], and these results could explain the greater presence of EVs in stressed medium. The reduction of sizes and concentrations of EVs in EEC medium on day 11 could be explained by the presence of cells that have survived the stress or that have had the ability to react to the stress by returning to near-physiological conditions.

The EV size and concentration within this co-culture system model increase our knowledge of endometritis because EVs may be transferring inflammatory signals.

## 5. Conclusions

Despite the interesting results obtained, all our studies were carried out in vitro. Embryos obtained through in vitro-assisted reproduction techniques present numerous discrepancies compared to embryos obtained in vivo [57], such as a significantly lower development rate [58] and a different miRNA cargo [59]. The goal of this research was to mimic the embryo––uterine environment stressed by an inflammatory process and develop a co-culture model with bidirectional communication between endometrial cells and embryos. The work in progress could be to study the proteomic profile and/or miRNA cargo of EECs and embryos in this experimental condition and, mainly, include an in vivo control to better understand this pathology.

It is possible to conclude that this in vitro model could represent an ideal tool to mimic endometrial stress and that EVs could represent a noninvasive tool for assessing the quality of bovine embryos cultured in vitro.

## Figures and Tables

**Table 1:** Viability values of EECs cultured in IVC for 11 days.

Time (Days)	% Viability (IVC/DMEM)
4	89.59 ± 5.56 ^a^
8	85.28 ± 8.83 ^a^
11	90.77 ± 6.12 ^a^

Legend: same superscript letters indicate no statistically significant differences (*p* > 0.05).

**Table 2:** Rate of embryos at different days of development in diverse embryo culture conditions.

Day of Culture	Embryo (% Blastocysts)	EECs+Embryo (% Blastocysts)	EECs+LPS+Embryo (% Blastocysts)
7	69/208 = 33.17 ± 2.54 ^a^	58/166 = 34.94 ± 1.95 ^a^	119/360 = 33.06 ± 3.08 ^a^
9	71/192 = 36.98 ± 3.22 ^a^	61/182 = 33.52 ± 3.22 ^a^	47/196 = 23.98 ± 3.41 ^b^
11	41/164 = 25.00 ± 2.10 ^a^	35/152 = 23.03 ± 3.18 ^a^	0/166 = 0 ^b^

Legend: different superscript letters indicate statistically significant differences in the lines.

## Data Availability

The original contributions presented in this study are included in the article/Supplementary Materials. Further inquiries can be directed to the corresponding author.

## Supplementary Materials

The following supporting information can be downloaded at https://www.mdpi.com/article/10.3390/ani16010038/s1: Figure S1: Stain-free images of full-length SDS-PAGE gel (A, left) and nitrocellulose membrane immediately post-transfer (B, right). The sample (S) was loaded in duplicate, adjacent to the molecular weight marker (M). Following transfer, the membrane was sectioned longitudinally and horizontally to enable independent incubation for each biomarker. Legend: “A. stain-free gel; B. stain-free membrane after transblot; M. marker; S. sample”. Figure S2: Row tiff image of Alix marker. First lane: marker (250-150-100-75 KDa); second lane: sample. Figure S3: Row tiff image of CD63 marker. Membrane on the left corresponds to picture in Figure 8A; membrane on the right is a replicate. First lane: marker (250-150-100-75-50-37 KDa); second lane: sample. Figure S4: Row tiff image of TSG101 marker. First lane: marker (50-37-25 KDa); second lane: sample.

## Abbreviations

The following abbreviations are used in this manuscript:

CCOs	cumulus oocyte complex
EECs	epithelial endometrial cells
Evs	extracellular vesicles
Gal-9	Galectin-9
HG-DMEM	Dulbecco’s Modified Eagle’s Medium high glucose
IVC	in vitro culture
IVF	in vitro fertilization
IVM	in vitro maturation
LIF	leukaemia inhibitory factor
LPS	lipopolysaccharide
MTT	tetrazolium dye 3-(4,5-dimethylthiazol-2-yl)-2,5-diphenyltetrazolium bromide
TBST	tris-buffered saline
TEER	trans epithelial electrical resistance
TEM	transmission electron microscopy
