# Supplementary material for: LPS-Stressed Bovine Endometrial Cells upon Morulae in a Transwell Model of Embryo—Maternal Talk
Source: Animals (Basel). 2025 Dec 23;16(1):38. doi: 10.3390/ani16010038 (PMC12785123; doi:10.3390/ani16010038)
Supplement: Supplementary file 1 [file animals-16-00038-s001.zip › animals-4044991-supplementary.pdf]

Supplementary Materials

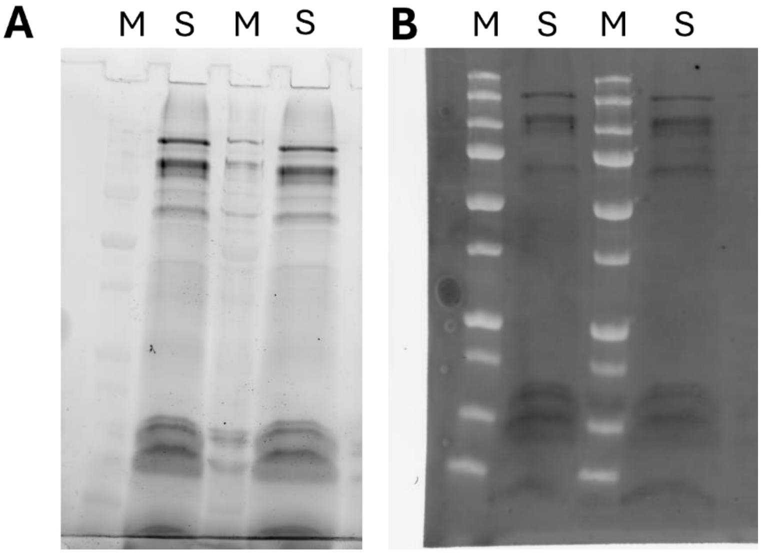

Figure S1. Stain-free images of full-length SDS-PAGE gel (A, left) and nitrocellulose membrane immediately post-transfer (B, right). The sample (S) was loaded in duplicate, adjacent to the molecular weight marker (M). Following transfer, the membrane was sectioned longitudinally and horizontally to enable independent incubation for each biomarker. Legend: "A. stain free gel; B. stain free membrane after transblot; M. marker; S. sample".

Figure S2. Row tiff image of Alix marker. First lane: marker (250-150-100-75 KDa); second lane: sample.

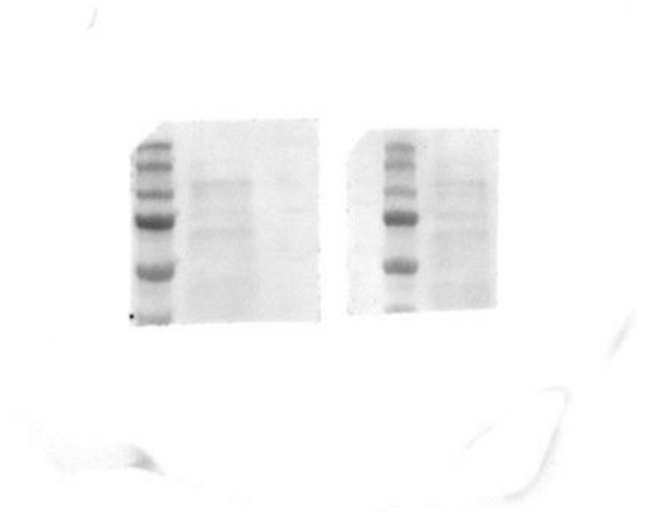

Figure S3. Row tiff image of CD63 marker. Membrane on the left corresponds to picture in Figure 8A; membrane on the right is a replicate. First lane: marker (250-150-100-75-50-37 KDa); second lane: sample.

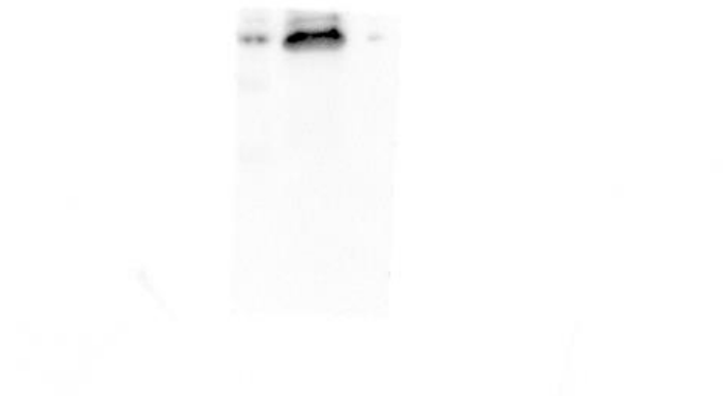

Figure S4. Row tiff image of TSG101 marker. First lane: marker (50-37-25 KDa); second lane: sample.
